# Direct measurement of stool consistency by texture analyzer and calculation of reference value in Belgian general population

**DOI:** 10.1038/s41598-021-81783-7

**Published:** 2021-01-27

**Authors:** Kazunori Matsuda, Takuya Akiyama, Satoshi Tsujibe, Kaihei Oki, Agata Gawad, Junji Fujimoto

**Affiliations:** 1 Yakult Honsha European Research Center for Microbiology VOF, Ghent-Zwijnaarde, Belgium; 2 Yakult Central Institute, Tokyo, Japan

**Keywords:** Constipation, Diarrhoea, Digestive signs and symptoms

## Abstract

Stool consistency is evaluated mainly in reference to indirect indicators such as water content or the appearance of stool forms using Bristol Stool Form Scale (BSFS). Methods of measurement are limited. We thus aimed to develop a simple protocol for direct measurement of stool consistency using the TA.XTExpress Texture Analyser (Stable Micro Systems Ltd.). We developed a protocol which enables mechanical quantification of the gram-force against a cylindrical probe (ø 6 mm) pushed into the stool surface at 2.0 mm/s to 5 mm depth. The consistency of 252 stools collected from 40 healthy Belgians was evaluated by the direct method and by the indirect indicators (water content and BSFS) for comparison. The log-transformed stool consistency values measured by the texture analyzer had a negative linear correlation with the stool water contents (*r*_*rm*_ = − 0.781) with homoscedastic variance, suggesting the appropriateness of the new protocol. They showed a similar correlation with the BSFS, but with a large variance in the consistency values of normal stool forms. This correlation was much smaller for BSFS scored by subjects (*r*_*rm*_ = − 0.587) than by experts (*r*_*rm*_ = − 0.789), collectively indicating BSFS as a rough indicator of stool consistency susceptible to subjective bias despite its effectiveness in clinical use. The optimized direct method using the texture analyzer enables the accurate quantification of stool consistency, which facilitates understanding of the intestinal environment and function and thus may enhance the value of the stool as a predictor of human health.

## Introduction

Having regular bowel movements is considered to be a sign of a healthy digestive system. Stool consistency strongly relates to stool transit time and defecation frequency, and therefore is regarded as a suitable indicator of bowel function. Irregular bowel movements (too frequent or infrequent) and repeated passages of hard or loose stools reduce quality of life and can indicate functional constipation (FC) or irritable bowel syndrome (IBS)^1^. Therefore, regular bowel movements with normal stool condition are desirable for good health.

Stool consistency is evaluated by indirect measurements which evaluate stool form visually; the Bristol Stool Form Scale (BSFS) is the one most widely used in both clinical and research settings^2–5^. The BSFS is a Likert-scale used to classify stool forms into seven categories, and has been validated as a surrogate measure for gastrointestinal transit time^6–11^. The Rome Foundation recommends use of the BSFS for diagnosing FC and IBS according to the Rome IV criteria^12^, and the European Food Safety Authority regards it as a validated questionnaire for measuring stool consistency in their guideline for health claim^13^. Although the BSFS can be easily evaluated by subjects themselves, this indirect method involves a degree of inter- and intra-rater variance, and modifications to the BSFS or training in its use should be explored to improve its validity and reliability^14,15^.

Stool consistency generally refers to the rheology and viscosity of the stool, which can be measured directly with a penetrometer^16,17^ and a viscometer^18,19^. Besides these technologies, a texture analyzer, which is used to measure multiple characteristics of foods and other consumer products, such as hardness, crispness, fracturability, adhesiveness and extensibility^20,21^, has been used to analyze stool consistency^22^. Physical stool hardness measured by a texture analyzer strongly correlates well with stool water content and thus accurately reflects stool consistency. However, the method was validated mainly in constipated people and requires complicated procedures: e.g. stools must be measured just within a few hours after defecation, and must be measured in multiple lumps throughout the length of the specimen to calculate the median value.

Here, we optimized methods for the direct measurement of stool consistency by a texture analyzer (TA.XTExpress Texture Analyser: TAXT; Stable Micro Systems Ltd.) to increase its operational convenience. The validity of the method was evaluated against stool water content and BSFS score. We analyzed differences in stool consistency according to gender, stool symptoms, and defecation time period in the general Belgian population.

## Results

### Correlation of stool consistency by TAXT with stool water content

**Figure 1:**
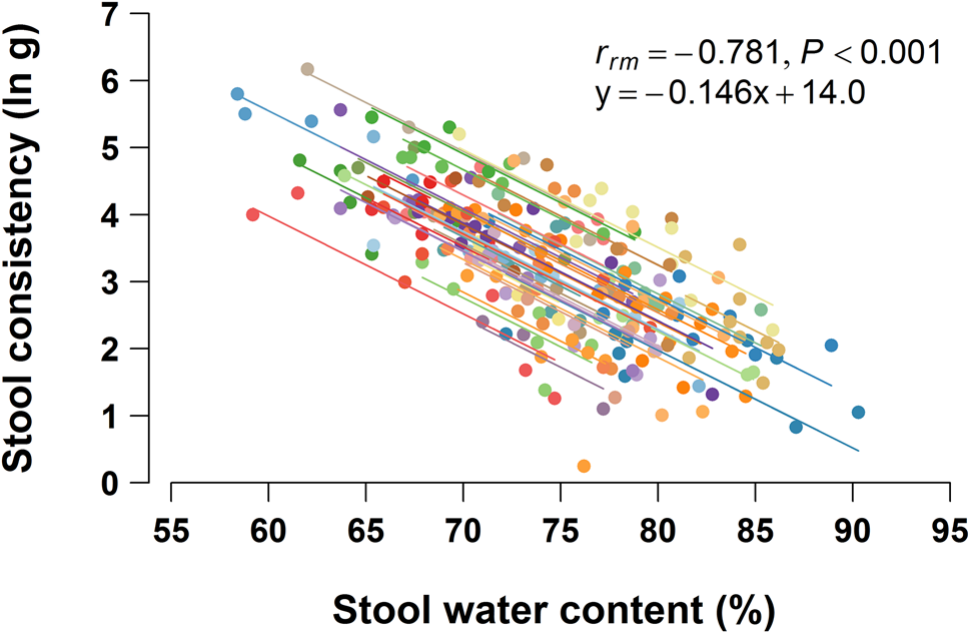
Correlation of stool consistency with stool water content. Repeated-measures correlation of stool consistency against stool water content was evaluated on 252 samples. Plots from the same subject were given the same color, and with corresponding line to show the best linear fit for each subject.

We optimized the methods of sample storage and preparation by conducting several preliminary experiments, and measured the consistency of 252 stool samples collected from 40 subjects. The natural-log-transformed (ln) stool consistency values (ln g/probe) were normally distributed (Shapiro–Wilk normality test, *P* = 0.595). Their mean value was 3.225 (95% CI 3.092 to 3.358; Fig. S1). The TAXT results had a strong negative correlation with the stool water contents, with *r*_*rm*_ = − 0.781 (95% CI − 0.828 to − 0.722; *P* < 0.001; Fig. 1). Variances across the variables was equal (homoscedastic).

### Comparison of stool consistency results with BSFS scores

**Figure 2:**
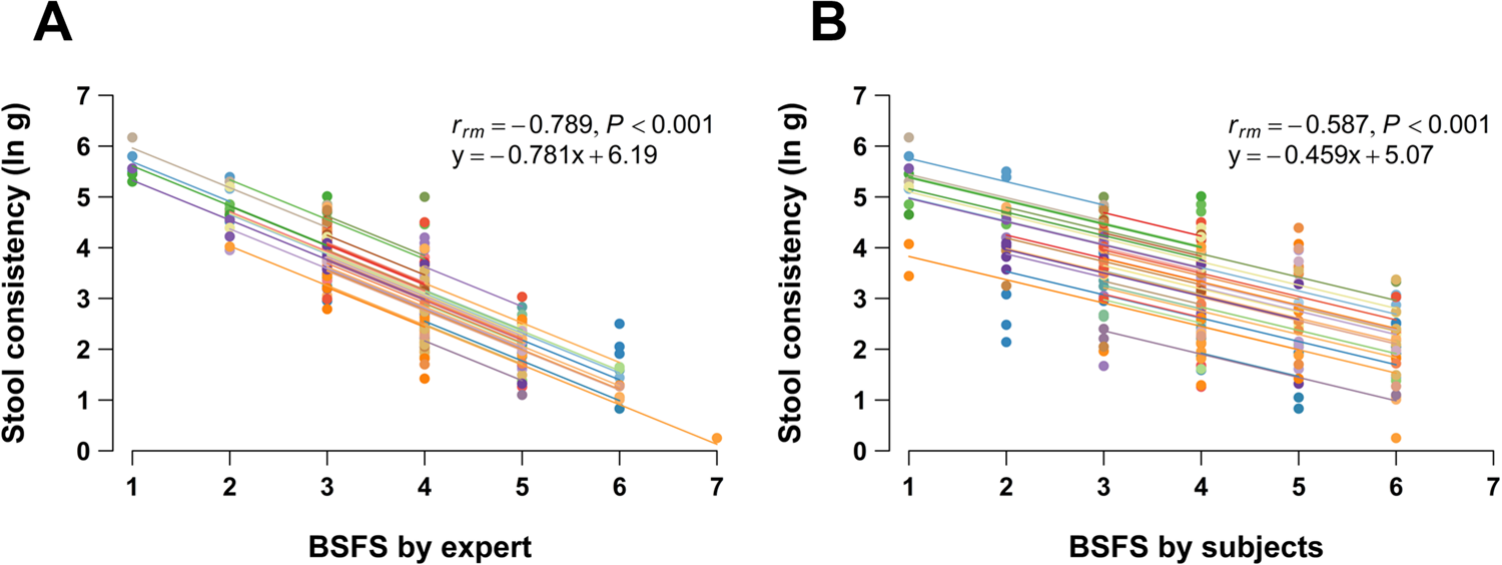
Comparison between BSFS scores and stool consistency. Repeated-measures correlation plots of 252 stool samples between stool consistency and BSFS score classified by (**A**) expert and (**B**) subjects. Plots from the same subject were given the same color, and with corresponding line to show the best linear fit for each subject.

The form of stool samples was rated on the BSFS by both the subjects and an expert in the laboratory. The stool consistency results from TAXT were negatively correlated with both BSFS scores (Fig. 2A,B), with significant *r*_*rm*_ values (*P* < 0.001). The correlation was much greater with the expert’s BSFS score (*r*_*rm*_ = − 0.789; 95% CI − 0.835 to − 0.732) than with the subjects’ (*r*_*rm*_ = − 0.587; 95% CI − 0.669 to − 0.491). The variances tended to depend on stool type, being greater for BSFS score 4 and the neighbor scores than at either ends.

### Stool consistency variation by gender, stool type, stool symptoms, and defecation time

The effect sizes of gender, stool type, stool symptoms, and time of defecation on the stool consistency estimated by linear mixed models showed no significant differences between genders in any category of stool types (hard, normal, soft) (Table 1). Stool consistency was significant for presence/absence of straining (estimate, 0.629; 95% CI 0.323 to 0.935; *P* < 0.001). This means that the average consistency (ln g) of the stools passed with straining is estimated to be 0.629 units higher (1.88-fold harder) than that of stools passed without straining. Gender had an interaction (*P* = 0.037): the estimate of straining was significant only in the woman’s subgroup (estimate, 0.889; 95% CI 0.479 to 1.299; *P* < 0.001). Stools passed in the morning tended to be softer than stools passed later (estimate, 0.211; 95% CI − 0.019 to 0.440; *P* = 0.073).

**Table 1:** Stool consistency variation according to gender, stool type, stool symptoms, and defecation time.

	Total	Woman (*N* = 22)	Man (*N* = 18)
All stool samples	3.225 ± 1.070 (252)	3.286 ± 1.099 (119)	3.171 ± 1.045 (133)
**Stool type based on Rome IV classification**			
Hard stool (BSFS 1 or 2)	4.956 ± 0.593 (21)	5.030 ± 0.677 (10)	4.889 ± 0.530 (11)
Normal stool (BSFS 3, 4, 5)	3.176 ± 0.877 (217)	3.227 ± 0.917 (103)	3.129 ± 0.840 (114)
Soft stool (BSFS 6 or 7)	1.394 ± 0.562 (14)	1.387 ± 0.224 (6)	1.399 ± 0.742 (8)
**With straining during evacuation**			
Yes	3.820 ± 1.039 (43)***	3.836 ± 0.834 (29)***	3.788 ± 1.410 (14)
No	3.103 ± 1.037 (209)	3.108 ± 1.119 (90)	3.098 ± 0.976 (119)
**With sensation of remaining stool in the rectum after evacuation**			
Yes	3.324 ± 1.157 (81)	3.241 ± 1.137 (55)	3.499 ± 1.201 (26)
No	3.178 ± 1.027 (171)	3.324 ± 1.073 (64)	3.091 ± 0.993 (107)
**Time of bowel movement**			
6:00 or later but before 12:00	3.150 ± 1.071 (121)	3.205 ± 1.129 (55)	3.105 ± 1.026 (66)
Other time period	3.294 ± 1.069 (131)	3.355 ± 1.077 (64)	3.236 ± 1.067 (67)

Stool consistency (ln g) is expressed as mean ± SD of results of stools in each category or subgroup. The effect size of each factors (gender, stool type, stool symptoms, and defecation time period) on stool consistency was evaluated by linear mixed models.

***P < 0.001 (between Yes and No).

## Discussion

We optimized sample storage and preparation to enhance the efficiency of the method for direct measurement of stool consistency by the texture analyzer. The log-transformed stool consistency had a strong negative linear correlation (*r*_*rm*_ = − 0.781) with stool water content, widely used as a surrogate indicator of stool consistency, and homoscedasticity (Fig. 1). The texture analyzer could detect the consistency of loose stools of BSFS types 6 and 7, with a water content of almost 90%, with high sensitivity. The stool consistency results had strong linear associations with every stool types (Fig. 2A). These results show that the new protocol provides accurate results of stool consistency for a wide variety of stool types. The texture analyzer has been widely used to measure multiple characteristics of foods and other consumer products. Owing to wide availability and high usability of the device in addition to simplicity of our improved protocol, the method can be readily introduced not only in laboratories for research use but also in general practitioners for the purpose to diagnose patients’ stool condition.

Other studies have reported that stool water content is correlated with stool hardness measured directly by a penetrometer and a texture analyzer^22,23^; our result is consistent with those (Fig. 1). Water content is obviously a major determinant of stool consistency, but several other factors have been proposed, such as the water-holding capacity of insoluble solids, as a higher ratio of water-insoluble to -soluble solids increased stool consistency^22^. Steatorrheas, with a higher content of soluble solids due to malabsorption of soluble dietary constituents (carbohydrates, proteins, and fatty acids), had a looser stool consistency than less fatty stools with the same stool water content^18^. Insoluble calcium fatty acid soaps (formed from calcium and fat, especially the long-chain fatty acids in the intestine) increase stool hardness, mainly in childhood population^24^. These other factors affect stool consistency to an extent, in addition to stool water content, and thus caused variability between stool consistency and water content.

The expert’s BSFS score had a strong correlation with stool consistency (Fig. 2A) and had a comparable *r*_*rm*_ score to that of stool consistency versus stool water content (Fig. 1). Thus, the BSFS offers a reliable surrogate measure of stool consistency when rated by a well-trained expert. However, BSFS scores of 3–5 had greater variance than the scores at either end. We assume that motions rated 3–5 were likely to comprise multiple stool forms mixed together than motions rated 1–2 or 6–7, allowing rating errors when categorizing such mixed-form stools into one representative BSFS score.

Stool consistency had a much higher correlation with the expert’s BSFS score (*r*_*rm*_ = − 0.789) than the subjects’ (*r*_*rm*_ = − 0.587) (Fig. 2A,B). Similar results were shown in a previous comparison of stool water content with BSFS scores^25^. These weaker correlations of BSFS score by subjects indicate inter-rater variability of BSFS assessment. Rating accuracy could vary among raters evaluating very small amounts of stool^25^, and evaluating stools with a mixture of forms within the same event or lump^22^. We also observed that when the BSFS is evaluated by subjects, the classification could be affected by their sensations during or after defecation (Table S1). The expert classified 217 stools as normal, including 19 classified as hard and 25 as soft by the subjects. Those 19 stools classified as hard were accompanied by straining with a higher frequency than the stools which matched the classification, and the 25 classified as soft were with a lower frequency of straining (*P* = 0.015, Fisher's exact test for 3 × 2 contingency table). This observation indicates that a sensation of straining during defecation influences subjective but not objective rating results. Such intra- and inter-variability can be eliminated by using a texture analyzer, which provides a highly accurate measurement of stool consistency.

We found no statistically significant differences in stool consistency between genders, although the women had slightly higher values than the men (Table 1). Nakaji et al. compared penetrometer-measured stool consistency between genders, and got similar results^17^. Women are slightly more likely than men to get constipation owing to several risk factors, including life style, hormones, and physical factors^26–28^. Therefore, their slightly higher stool consistency could be a result of longer transit time in these women. Stools passed in the morning tended to be softer than those passed later (Table 1). Normal colonic motility involves coordination by the neural circuitry of colonic contraction in response to meals and the diurnal cycle^29^. Colonic contractions slow during sleep and resume after awakening, often resulting in a bowel movement^29,30^. If instead a bowel movement does not occur after awakening in the morning and is delayed, the stool becomes harder owing to the longer transit time in the intestine. Therefore, it seems reasonable that stools passed in the morning are softer. This colonic wake response is impaired in patients with chronic constipation and slow transit^31,32^. So subjects who typically defecate in the morning can be regarded as being at a lower risk for constipation.

Both hard and loose stools are signs of a gut or digestive problem. Persisting production of such stools results in difficult, painful, infrequent or perceived incomplete evacuation of bowel movements, indicating FC or IBS. Disorders of gastrointestinal function cause a significant decline in quality of life, and therefore several treatments such as lifestyle changes^33^, dietary modifications (including supplementation with probiotics^34,35^ and fibre^36^), medications, and therapeutic modalities^37^ are used to relieve symptoms. When we evaluate the effects of interventions on the improvement of stool consistency, a reference value must be decided. We extracted the 217 stool samples classified as normal (BSFS types 3–5) by the expert according to the Rome IV diagnostic criteria and calculated their average value (3.176, ln g/probe) as the reference value of a normal stool. There was no significant difference between genders (Table 1) or among ages (data not shown), so this reference value can be applied to any population. Although further studies are needed to confirm its applicability, we can now quantitatively evaluate the efficacy of treatments by examining changes in absolute differences from this reference value for every stool sample.

In summary, direct measurement of stool consistency with a texture analyzer provided accurate results for wide variety of stool types. It agreed well with the conventional BSFS, but the accuracy of BSFS classification was affected by various factors, including inter-rater variability and subjects’ sensations during defecation. The direct measurement of stool consistency at high resolution and high accuracy will better explain the relationships of stool consistency with diseases, dietary and nutrient intakes, and gut microbiota composition^2^. We propose a reference value for a normal stool, based on a Belgian population, with which to evaluate the effects of interventions on the improvement of stool condition. Further data accumulation is necessary to confirm its applicability.

## Methods

### Study design and subjects

We collected stool samples to validate a method for the direct measurement of stool consistency. The study was conducted in Belgium during June and July 2017 as an interventional study without provision of treatment. A minimum of 120 stool samples had to be analyzed to ensure a sufficient amount of samples of each BSFS category for a proper validation of the method. Forty subjects were selected such that the widest possible variety in consistency of stool samples could be obtained, under the assumption that at least three stool samples would be provided per subject. For pre-screening, a questionnaire about usual stool pattern was given to 100 healthy adults (52 women, 48 men aged ≥ 18 years) living in Flanders. The subjects specified the percentage of their usual stool pattern classified into the following three categories: BSFS types 1–2 (hard), scored as 1; 3–5 (normal), scored as 2; and 6–7 (soft), scored as 3 (Fig. S2). The corresponding percentages were multiplied with these scores, and the sum was considered as the usual stool pattern score. Forty subjects were randomly selected through stratification according to gender and usual stool pattern score to achieve the widest possible variety in consistency of stool samples, and finally 22 women and 18 men were enrolled (average age ± SD, 37.7 ± 12.2 years; min. 22, max. 69). The study set two stool collection periods of approximately 60 h each, starting on Friday evening and ending on Monday morning of 2 consecutive weeks. The subjects were instructed to collect every stool produced during the periods.

### Stool specimens

The 40 subjects collected 252 bulk stool samples. The specimens were collected in a Commode Specimen Collection System (catalog no. DYND36500; Medline Industries, Inc., Illinois, USA), cooled in a portable refrigerator at the subjects' home and kept cold during transport to the laboratory. The storage time from sampling to analysis varied among samples (average ± SD, 41.8 ± 20.3 h; min. 2.5, max. 91.1). In a preliminary experiment, we assessed the effect of storage on stool consistency. The consistency values of specimens stayed the same throughout 6 days’ storage, with negligible variation (Fig. S3), with a coefficient of variance (CV = 2.57: average of three subjects) smaller than the measurement error (CV = 3.00). We therefore used all stool samples in the analysis.

The subjects recorded information concerning each stool in a diary: date and time of defecation, form of the stool (according to the BSFS), presence/absence of straining, and presence/absence of sensation of remaining stool in the rectum.

### Measurement of stool consistency

**Figure 3:**
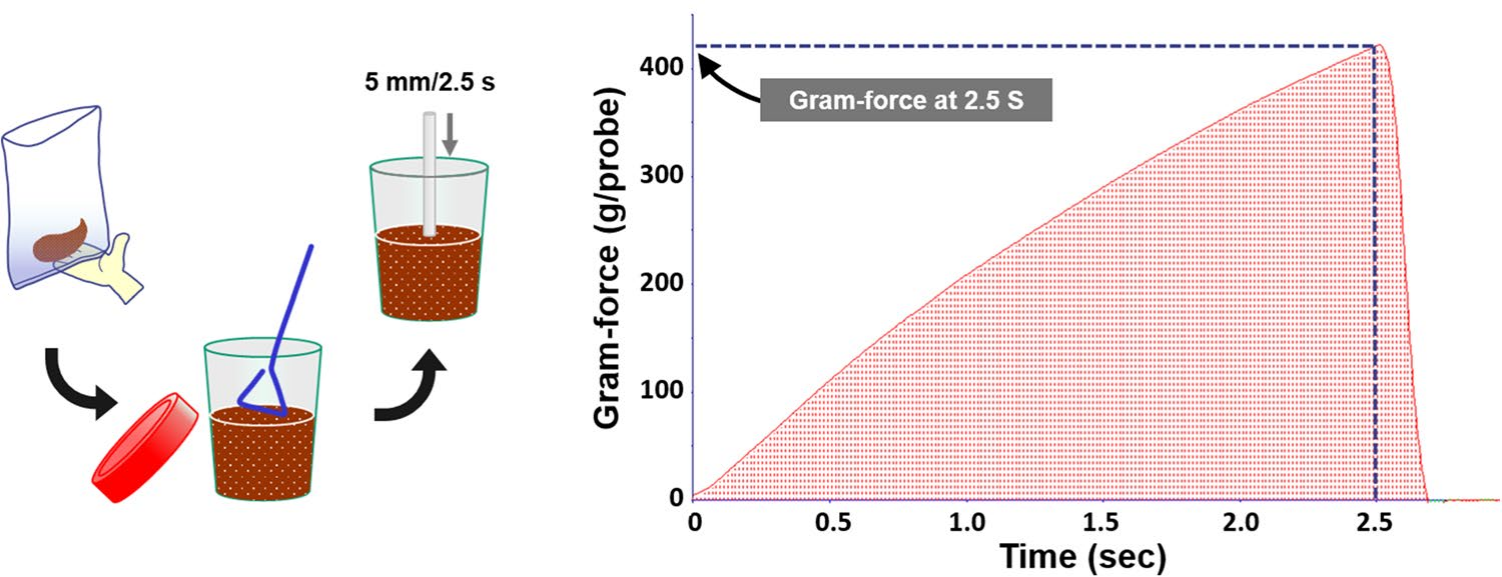
Schematic view of stool consistency measurement by texture analyzer. Whole stool was homogenized by hand in a plastic bag. A portion was placed in a plastic container and flattened with a plastic spreader. A cylindrical stainless steel probe (ø 6 mm) moved downward at a constant 2.0 mm/s, and the gram-force against the probe was monitored until the probe reached 5 mm down from the surface. The gram-force at the inflection point was measured as an outcome of stool consistency.

We used a TA.XTExpress Texture Analyser (TAXT; Stable Micro Systems Ltd., Godalming, UK) to measure stool consistency. Each whole stool was transferred into a plastic bag and was homogenized for 30 s by hand (Fig. 3). A portion was placed into a plastic container (cat. no. 75.562.105; Sarstedt AG & Co. KG, Nümbrecht, Germany), which was stored in a refrigerator. Since we earlier observed that stool consistency increased after refrigerated storage, we assessed how to restore it by returning the refrigerated stools to room temperature (20–25 °C) and leaving them for 30, 60 or 120 min, and found that it returned almost to the original after 30 min (data not shown). Thus, we took the plastic containers with the homogenized stool out from the refrigerator and kept them at room temperature for 30 min before measurement.

The surface of each homogenized stool was flattened with a plastic spreader. A cylindrical stainless steel probe 6 mm wide was set above it and descended downward at a constant speed (2.0 mm/s), and the gram-force against the probe (g/probe) and the elapsed time were recorded in EXPONENT Lite Express v. 6.1.11.0 software (Stable Micro Systems Ltd.) during the compression until the probe reached 5 mm down from the surface. From the force–time curve (Fig. 3), the gram-force at 2.5 s was used as an outcome of stool consistency. The gram-force was measured five times at different points, and the average without the lowest and highest values was calculated so as to eliminate artifacts such that the probe hits undigested food particles and air space.

### Stool water content

Stool water content was determined by lyophilization. After homogenization as above, 4.5–5.5 g of homogenized stool was put into a plastic tube (cat. no. 80.734.001; Sarstedt AG & Co. KG) and weighted before freezing at − 20 °C. After lyophilization, the stool water content (%) was determined from the difference in weight.

### BSFS classification

The BSFS defines seven categories of stool form (Fig. S2). Subjects chose the category most like their stools at every defecation and recorded the score in the diary. An expert also scored each stool in the laboratory.

### Statistical analysis

Statistical analyses were performed in R v. 3.4.1 software. For correlation analysis, since each subject provided multiple stool samples, repeated-measures correlation using the rmcorr package^38^ was applied to account for non-independence among observations, and a repeated-measures correlation coefficient (*r*_*rm*_), *P* value, and a 95% confidence interval (CI; computed analytically using the Fisher transformation) for *r*_*rm*_ were calculated. R packages lme4 and lmerTest were used for linear mixed model analysis. The models used the stool consistency as the response variable; a sole explanatory variable of gender, stool type, stool symptoms, or defecation time of day; and a random term for individual. The fixed effect size of each explanatory variable was estimated in total stools and in gender subgroups. All statistical tests were two-sided and were performed at the 0.05 level of significance. A *P* value of ≥ 0.05 and < 0.10 was considered to indicate a trend towards significance. All *P* values were rounded to three decimal places and are presented as “ < 0.001” if they were < 0.001 after rounding. Owing to the exploratory character of the study and the clear definition of one primary outcome parameter, no correction for multiplicity testing was applied. There was no missing value in all the observations on the collected 252 stool samples.

### Ethical issues

The ethics committee of Onze-Lieve-Vrouw Hospital Aalst approved the study protocol (B126201731617), and all participants gave their informed consent. The study protocol was registered at ClinicalTrials.gov (registration number NCT03188302). The trial was conducted in compliance with the International Conference on Harmonization-Good Clinical Practice (ICH GCP) guidelines and applicable regulatory requirements, and in accordance with the Declaration of Helsinki in its most recent version.

## Supplementary Information

Supplementary Information.
