# Supplementary material for: Direct measurement of stool consistency by texture analyzer and calculation of reference value in Belgian general population
Source: Sci Rep. 2021 Jan 27;11:2400. doi: 10.1038/s41598-021-81783-7 (PMC7840686; doi:10.1038/s41598-021-81783-7)
Supplement: Supplementary file 1 — Supplementary Information. [file 41598_2021_81783_MOESM1_ESM.pdf]

Supplementary Information for

Direct measurement of stool consistency by texture analyzer and calculation of reference value in Belgian general population

Kazunori Matsuda[1,2]✉, Takuya Akiyama[1,2], Satoshi Tsujibe[1], Kaihei Oki[1,2], Agata Gawad[1], and Junji Fujimoto[1,2]

[1]Yakult Honsha European Research Center for Microbiology VOF, Technologiepark 94 bus 3, 9052 Ghent-Zwijnaarde, Belgium. [2]Yakult Central Institute, 5-11 Izumi, Kunitachi-shi, Tokyo, 186-8650 Japan. ✉email: kazunori-matsuda@yakult.co.jp

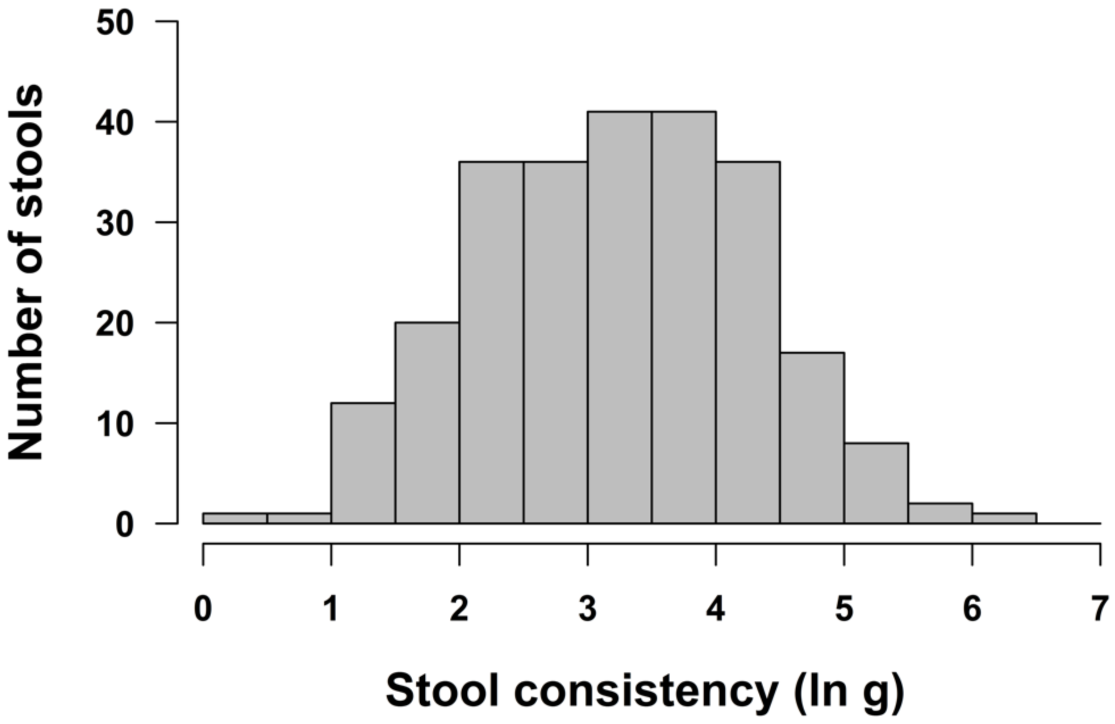

Figure S1. Distribution of consistency values of stool samples from healthy Belgian adults. Histogram of log-transformed stool consistency values (ln g/probe). Values of 252 stool samples collected from 40 subjects were measured by texture analyzer.

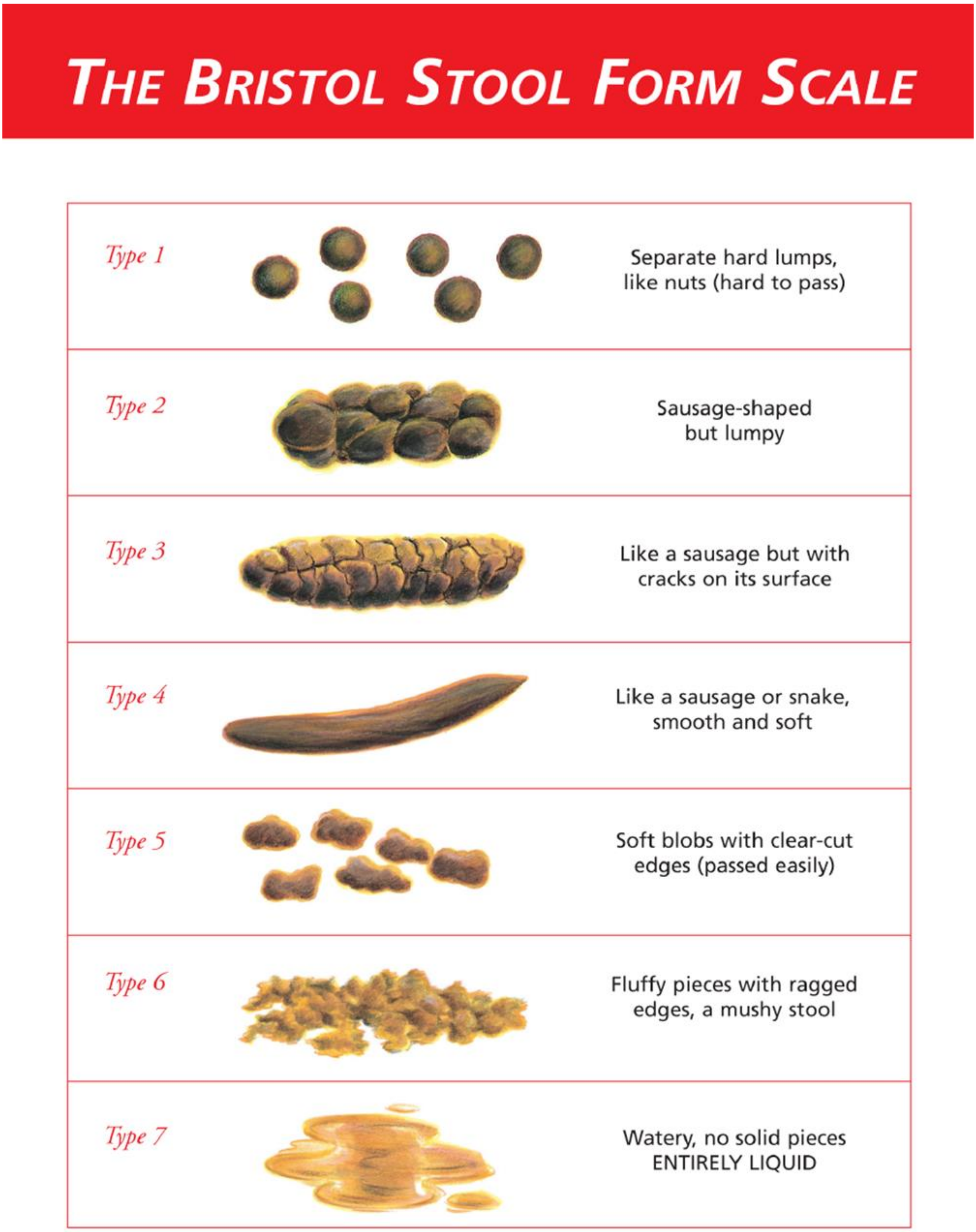

Figure S2. The Bristol Stool Form Scale. Reproduced by kind permission of Dr. KW Heaton, Reader in Medicine at the University of Bristol. ©2000 Produced by Norgine Pharmaceuticals Limited.

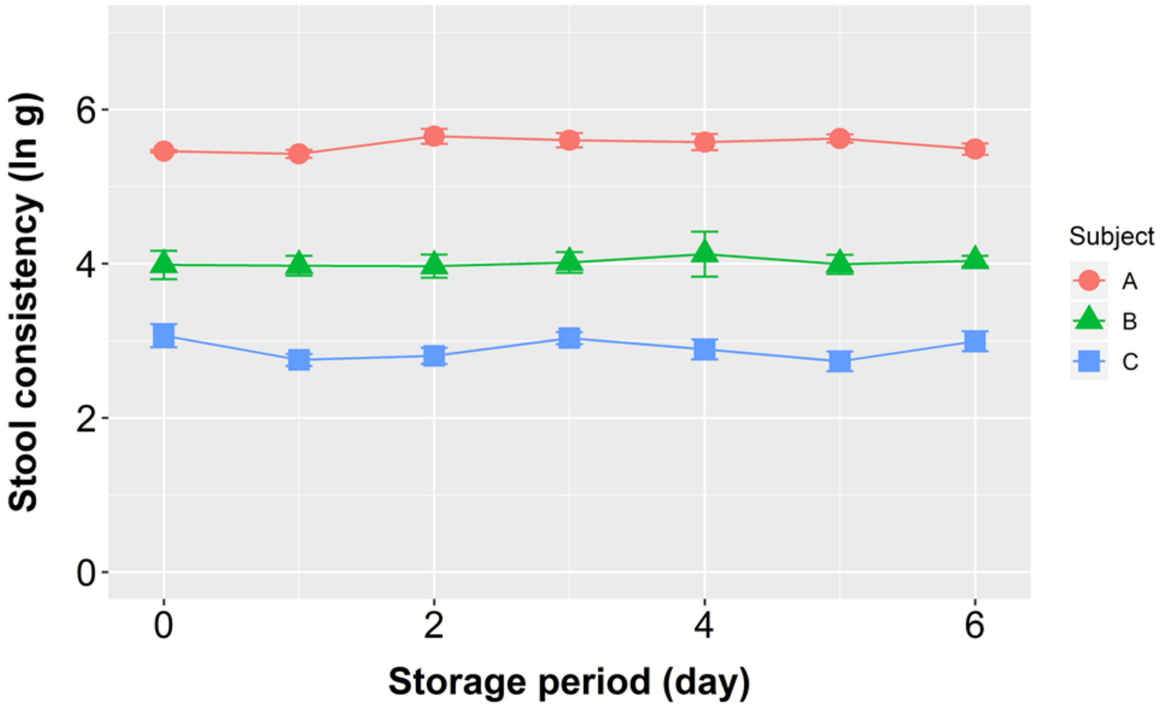

Figure S3. Change of stool consistency during refrigerator storage. Stool specimens were collected from 3 subjects (A, B, C) and their consistency (ln g) was repeatedly measured by texture analyzer just after defecation (day 0) and during refrigerator storage for 6 days.

Table S1. Agreement of stool type classification between raters and its relations with stool symptoms.

A. Straining

Stool type classified by subjects	Stool type classified by expert (actual classification)		
	Hard/lumpy	Normal	Loose/watery
Hard/lumpy	33.3% (5/15)	36.8% (7/19)	–
Normal	16.7% (1/6)	16.2% (28/173)	0.0% (0/6)
Loose/watery	–	4.0% (1/25)	12.5% (1/8)

B. Incompleteness

Stool type classified by subjects	Stool type classified by expert (actual classification)		
	Hard/lumpy	Normal	Loose/watery
Hard/lumpy	40.0% (6/15)	42.1% (8/19)	–
Normal	66.7% (4/6)	31.8% (55/173)	16.7% (1/6)
Loose/watery	–	20.0% (5/25)	25.0% (2/8)

Cells shading denotes classification by subjects that agree with classification by expert. Frequencies of sensation of (A) straining and (B) incompleteness during/after defecation are shown. –, not applicable.
